# Transplant Centers That Measure Frailty as Part of Clinical Practice Have Better Outcomes

**DOI:** 10.1093/geroni/igab046.2050

**Published:** 2021-12-17

**Authors:** Xiaomeng Chen, Yi Liu, Nadia Chu, Jeremy Walston, Dorry Segev, Mara McAdams-DeMarco

**Affiliations:** Johns Hopkins University School of Medicine, Baltimore, Maryland, United States

## Abstract

Frailty predicts adverse outcomes for kidney transplant (KT) patients; yet the impact of clinical assessments of frailty on center-level outcomes remains unclear. We sought to test whether KT centers that measure frailty as part of clinical practice have better pre- and post-KT outcomes. We conducted a survey of US transplant centers (11/2017-4/2018), 132 KT centers (response rate=65.3%) reported frequencies of frailty assessment at candidacy evaluation and KT admission. Center characteristics and clinical outcomes were gleaned from the national registry (2017-2019). Poisson regression was used to estimate incidence rate ratios (IRRs) of waitlist mortality rate and transplantation rate in candidates and graft loss rates in recipients by frequency of frailty assessment. All models were adjusted for case mix and center characteristics. Given similar center characteristics, centers assessing frailty at evaluation had a lower waitlist mortality rate (always=3.5, sometimes=3.2, never=4.1 deaths per 100 person-years). After adjustment, centers assessing frailty at evaluation had a lower rates of waitlist mortality (always IRR=0.91, 95% CI:0.84-0.99; sometimes=0.89, 95% CI:0.83-0.96) and transplantation (always IRR=0.94, 95% CI:0.91-0.97; sometimes=0.88, 95% CI:0.85-0.90) than those never assessing frailty. Centers that always assessed frailty at KT admission had 0.71 (95% CI:0.54-0.92) times the rate of death-censored graft loss than their counterparts never assessing frailty. Assessing frailty at evaluation is associated with lower transplantation rate but better waitlist survival; centers always assessing frailty at admission are likely to have better graft survival. Research is needed to explore how routine assessment of frailty in other clinical practices benefits broader patient populations.

